# Supplementary material for: FcγRIIB-I232T polymorphic change allosterically suppresses ligand binding
Source: eLife. 2019 Jul 25;8:e46689. doi: 10.7554/eLife.46689 (PMC6711707; doi:10.7554/eLife.46689)
Supplement: Supplementary file 1. [file elife-46689-supp1.docx]

**Association analysis of rs1050501 with SLE (adjusted for sex and age)**

| **rs1050501** | **Control** | **SLE** | **OR** | **95% CI** | ***p* value** |
| --- | --- | --- | --- | --- | --- |
| **allelic** |  |  |  |  |  |
| **T** | 1038 (75.4) | 1039 (73.1) | 1.142 | 0.958-1.362 | 0.138 |
| **C** | 338 (24.6) | 383 (26.9) |  |  |  |
| **genotypic** |  |  |  |  |  |
| **TT+TC** | 662 (96.2) | 661 (92.7) | 1.927 | 1.185-3.134 | 0.008 |
| **CC** | 26 (3.78) | 50 (7.03) |  |  |  |
