# Supplementary material for: FcγRIIB-I232T polymorphic change allosterically suppresses ligand binding
Source: eLife. 2019 Jul 25;8:e46689. doi: 10.7554/eLife.46689 (PMC6711707; doi:10.7554/eLife.46689)
Supplement: Supplementary file 2. [file elife-46689-supp2.docx]

**Demographic characteristic of SLE cohort**

| Characteristics | Controls  (n = 688) | SLE cases  (n = 711) |
| --- | --- | --- |
| Female (%) | 90.6 | 90.6 |
| Age (mean ± SD years) | 36.1 ± 11.4 | 36.0 ± 13.2 |
| Age of onset (mean ± SD years) |  | 30.5 ± 12.1 |
| Disease duration (mean ± SD years) |  | 5.5 ± 6.4 |
| SLEDAI [median (IQR)] |  | 6 (0~24) |
| Clinical manifestations (%) |  |  |
| LN (n = 289) |  | 57.1 |
| NPSLE (n = 286) |  | 10.1 |
| Rash (n = 605) |  | 67.4 |
| Photosensitivity (n = 593) |  | 34.7 |
| Oral ulcers (n = 589) |  | 31.6 |
| Raynaud (n = 578) |  | 29.8 |
| Arthritis (n = 630 |  | 63.3 |
| Leukopenia (n = 533) |  | 52.0 |
| Thrombopenia (n = 521) |  | 30.5 |
| Complement depressed (n = 538) |  | 78.3 |
| Autoantibody positivity (%) |  |  |
| ANA (n = 632) |  | 93.0 |
| Anti-dsDNA (n = 615) |  | 61.6 |
| Anti-Ro (SSA) (n = 591) |  | 46.7 |
| Anti-La (SSB) (n = 553) |  | 17.7 |
| Anti-Sm (n = 564) |  | 26.2 |
| Anti-r-NRP (n = 555) |  | 31.0 |
| AHA (n = 322) |  | 19.9 |

SLE: systemic lupus erythematous; SLEDAI: SLE Disease Activity Index; LN: lupus nephritis; NPSLE: neuropsychiatric SLE; ANA: antinuclear autoantibodies; Anti-dsDNA: anti-double stranded DNA antibodies; Anti-Ro (SSA): Anti-Ro (SSA) antibody; Anti-La (SSB): Anti-La (SSB) antibody; Anti-Sm: anti-Smith antibody.
